# Characterization of a new N-terminally acetylated extra-mitochondrial isoform of frataxin in human erythrocytes

**DOI:** 10.1038/s41598-018-35346-y

**Published:** 2018-11-19

**Authors:** Lili Guo, Qingqing Wang, Liwei Weng, Lauren A. Hauser, Cassandra J. Strawser, Clementina Mesaros, David R. Lynch, Ian A. Blair

**Affiliations:** 1 Penn SRP Center and Center of Excellence in Environmental Toxicology Center, Department of Systems Pharmacology and Translational Therapeutics Perelman School of Medicine, University of Pennsylvania, Philadelphia, PA 19104 United States; 2 Penn/CHOP Center of Excellence in Friedreich’s ataxia, Philadelphia, PA 19104 United States; 3 Departments of Pediatrics and Neurology, Children’s Hospital of Philadelphia, Philadelphia, PA 19104 United States; 4 Departments of Pediatrics and Neurology Perelman School of Medicine, University of Pennsylvania, Philadelphia, PA 19104 United States

## Abstract

Frataxin is a highly conserved protein encoded by the frataxin (*FXN*) gene. The full-length 210-amino acid form of protein frataxin (1–210; isoform A) expressed in the cytosol of cells rapidly translocates to the mitochondria, where it is converted to the mature form (81–210) by mitochondrial processing peptidase. Mature frataxin (81–210) is a critically important protein because it facilitates the assembly of mitochondrial iron-sulfur cluster protein complexes such as aconitase, lipoate synthase, and succinate dehydrogenases. Decreased expression of frataxin protein is responsible for the devastating rare genetic disease of Friedreich’s ataxia. The mitochondrial form of frataxin has long been thought to be present in erythrocytes even though paradoxically, erythrocytes lack mitochondria. We have discovered that erythrocyte frataxin is in fact a novel isoform of frataxin (isoform E) with 135-amino acids and an N-terminally acetylated methionine residue. There is three times as much isoform E in erythrocytes (20.9 ± 6.4 ng/mL) from the whole blood of healthy volunteers (n = 10) when compared with the mature mitochondrial frataxin present in other blood cells (7.1 ± 1.0 ng/mL). Isoform E lacks a mitochondrial targeting sequence and so is distributed to both cytosol and the nucleus when expressed in cultured cells. When extra-mitochondrial frataxin isoform E is expressed in HEK 293 cells, it is converted to a shorter isoform identical to the mature frataxin found in mitochondria, which raises the possibility that it is involved in disease etiology. The ability to specifically quantify extra-mitochondrial and mitochondrial isoforms of frataxin in whole blood will make it possible to readily follow the natural history of diseases such as Friedreich’s ataxia and monitor the efficacy of therapeutic interventions.

## Introduction

Frataxin is a highly conserved protein encoded by the frataxin gene (*FXN*), which can be found in both prokaryotes and eukaryotes^1,2^. Although the exact role of frataxin has not been completely delineated, there is compelling evidence that it is essential for iron-sulfur cluster biogenesis^3^. Frataxin is known to be involved in the formation of iron-sulfur cluster proteins in the mitochondrial compartment, such as lipoate synthase, aconitase, and succinate dehydrogenase^4^. It enhances sulfur transfer to ISCU, the scaffold protein on which iron-sulfur cluster proteins are assembled^5^, and accelerates a rate-limiting sulfur transfer step in the synthesis of [2Fe-2S] clusters^6^. Epigenetic silencing of the *FXN* gene and the resulting deficiency in frataxin protein is considered to be the biochemical defect responsible for the devastating effects observed in patients with Friedreich’s ataxia (FA)^2,7,8^.

The major frataxin (*FXN*) mRNA transcript in humans (*FXN*-1, 1.3 kb), is composed of five exons (1 A, 2–4, and 5 A). *FXN*-1 mRNA encodes the full-length 210-amino acid form of protein frataxin (1–210) with a molecular weight (MW) of 23,135 Da sometimes known as isoform A or *FXN-1* (Q16595–1, Uniprot)^1,9^. Full-length frataxin (1–210) protein is rapidly translocated from the cytosol to the mitochondria where it is cleaved by mitochondrial processing peptidase (MPP) at the R-2 sites (arginine present two amino acids towards the amino terminus)^10^. The mature biologically active form of frataxin (81–210) in mitochondria arises from a two-step process^11^. Initial MPP-mediated cleavage of full-length frataxin (1–210) occurs at the R-2 site between G^41^-L^42^ to give an intermediate form of frataxin (42–210), a 169-amino acid protein with a MW of 18,826 Da. This then undergoes a second MPP-mediated cleavage at K^80^-S^81^ (a second R-2 site) to give mature frataxin (81–210), as a 130-amino acid protein with a MW of 14,268 Da. If the K^80^-S^81^ site is blocked then cleavage can occur at a third R-2 site (A^55^-S^56^) to give another intermediate form of frataxin (56–210) as a 155-amino acid protein with a MW of 17,255 Da^11^. There is a report that Fe^II^ can induce cleavage of the full-length form of frataxin (1–210) at N^77^-L^78^ to give frataxin (78–210), a 133-amino acid protein with a MW of 14,665 Da, which does not appear to be formed *in vivo*^12^.

Four additional transcripts of the FXN gene have been reported^9,13–15^. A minor alternative transcript, FXN-2 mRNA contains exon 5B in place of exon 5A in FXN-1 mRNA. An in-frame stop codon in exon 5B of FXN-2 mRNA results in it encoding a shorter protein of 171-amino acids known as isoform B or FXN-2 (Q16595-2, Uniprot, MW = 19,095 Da)^9,13^. Another minor alternative transcript, FXN-3 mRNA, differs from FXN-1 mRNA through an insertion of 8 bp due to an alternative splice site at the 5′ end of intron 4. The 8 bp insertion generates a frameshift that introduces a stop codon site, so that this transcript encodes for a protein of 196-amino acids known as FXN-3 (Q16595-3, Uniprot, MW = 21,416 Da)^9,13^. These two shorter proteins, both contain the N-terminal mitochondrial targeting sequence^1^. Two additional transcripts were discovered in cell culture and human tissues, which could give rise to frataxin isoforms II and III that lack the N-terminal mitochondrial targeting sequence^14^. Isoform II transcript, which should produce a 135-amino acid form of frataxin (76–210), was found primarily in cerebellum. Isoform III transcript, which should produce a 164-amino acid form of frataxin (1–5, 53–210), was found primarily in the heart^14^. The biological relevance of these four additional forms of frataxin is not clear and no endogenous protein corresponding to these transcripts has ever been detected.

Surprisingly, mature frataxin (81–210), a mitochondrial protein, has been found previously in erythrocytes by both western blot and dipstick immunoassay^16,17^, although erythrocytes have no ribosomal machinery or mitochondria. There is no obvious role for erythrocyte frataxin because the ferrochelatase-mediated conversion of protoporphyrin IX to heme B catalyzed by frataxin occurs in mitochondra^18–20^ before the erythrocytes have formed. Little attention has been given to the paradoxical presence of this mitochondrial protein in erythrocytes, a cell with no mitochondria. This is most likely because aberrant heme formation is not observed in FA^21^ where frataxin levels in erythrocytes are significantly reduced from 70 ng/mL of whole blood from control subjects to 17 ng/mL in subjects with the disease^16^. We report that erythrocyte frataxin is in fact an N-terminally acetylated 135 amino acid splice variant of frataxin, which lacks the mitochondrial targeting sequence found in full-length frataxin. We also provide evidence that this N-terminally acetylated form of frataxin is a precursor of extra-mitochondrial mature frataxin that is normally found within the mitochondria.

## Results

### A unique form of frataxin exists in erythrocytes

**Figure 1:**
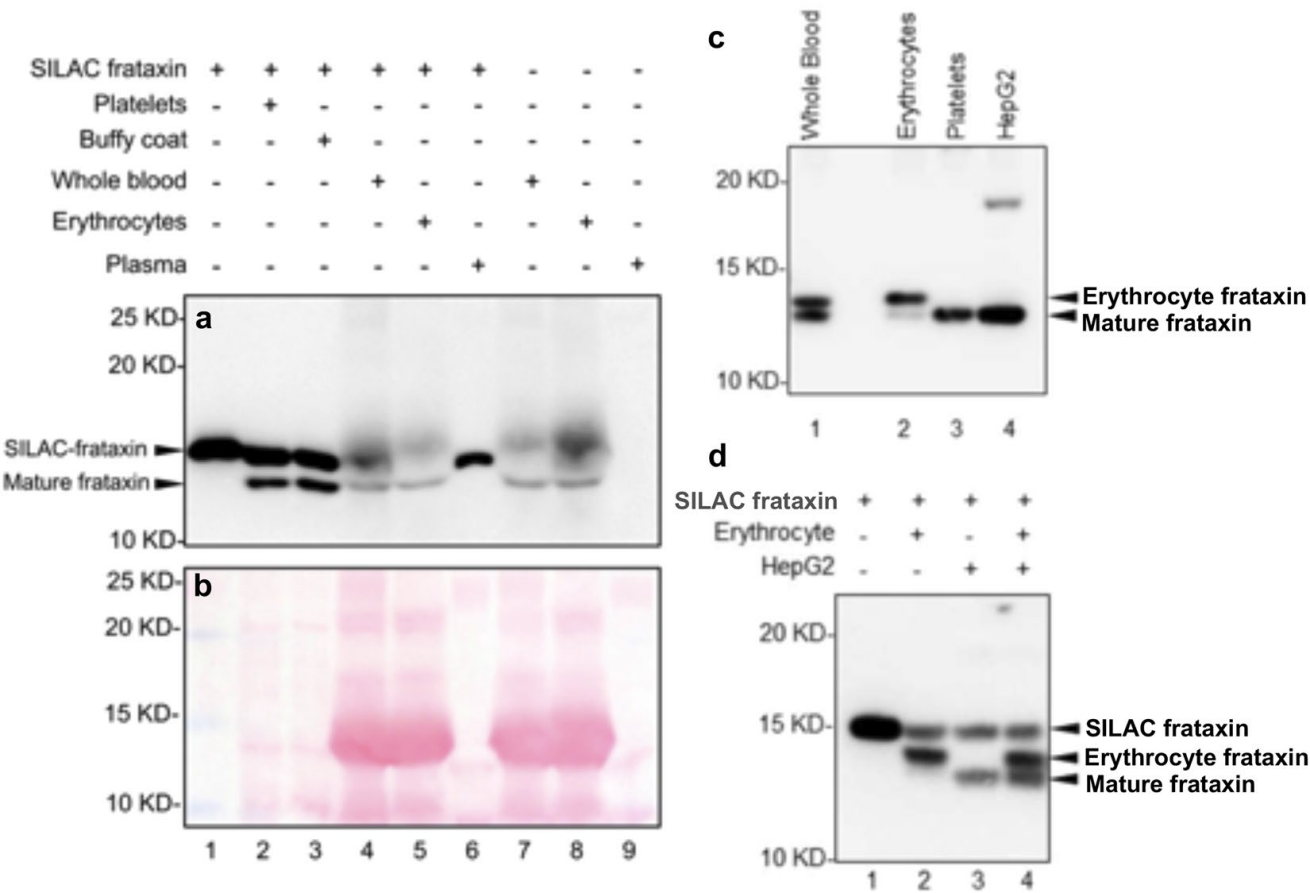
Frataxin from erythrocyte migrates slower in SDS-PAGE gel than the mature form after IP. (**a**) Platelets (lane 2), buffy coat (lane 3), whole blood (lane 4), erythrocyte (lane 5), and plasma (lane 6) lysates were analyzed after adding a 1 ng frataxin SILAC standard (lane 1). Whole blood (lane 7), erythrocytes (lane 8), and plasma (lane 9) lysates were also analyzed without the SILAC frataxin standard. Samples were filtered through a 50 kDa filter to remove abundant high molecular weight proteins before SDS-PAGE and western blot analysis. (**b**) As for (**a**) but with Ponceau staining. The presence of HBB in erythrocyte and whole blood interfered with the analysis of endogenous and SILAC labeled mature frataxin (lanes 4, 5, 7 and 8). High abundance proteins from the erythrocytes eluted with a similar mobility to mature frataxin. (**c**) Whole blood (lane 1), erythrocyte (lane 2), platelet (lane 3) and HepG2 (lane 4) lysates were analyzed after IP using anti-frataxin (1D9) mAb and blotted using another anti-frataxin mAb (Abcam). (**d**) Erythrocyte (lane 2), HepG2 cell (lane 3), Erythrocyte + HepG2 cell (lane 4) lysates were purified and analyzed as in (**c**) except that the SILAC frataxin standard was added to the lysates. The SILAC standard was added directly to lane 1.

The relatively low reported concentrations of frataxin in blood (70 ng/mL)^16^, the viscosity of the erythrocyte lysates, and the presence of high abundance proteins, such as 16.0 kDa human hemoglobin-β (HBB), made it difficult to accurately assess the migration of erythrocyte frataxin on SDS-PAGE by western blot analysis. A frataxin standard expressed through stable amino acid labeling by amino acids in cell culture (SILAC) containing a 6xHis tag^22^ (Fig. 1a, lane 1) was readily separated from platelet mature frataxin (Fig. 1a, lane 2), and buffy coat mature frataxin (Fig. 1a, lane 3). However, when erythrocytes were present, interference from erythrocyte proteins observed in the Ponceau stain (Fig. 1b) obscured the western blot when analyzed with the SILAC standard (Fig. 1a,b, lanes 4 and 5) or without the SILAC standard (Fig. 1a,b, lanes 7 and 8). In fact, cross-reactivity of the mAb with HBB appeared to show the presence of the SILAC standard even when it was not present (higher smeared bands in Fig. 1a,b, lanes 7 and 8). Therefore, it is unclear whether the bottom bands at the size of mature frataxin were endogenous frataxin or non-specific signals. In order to remove the interfering proteins observed on Ponceau staining (Fig. 1b), erythrocyte lysates or whole blood were subjected to IP using one anti-frataxin mAb (1D9, LSBio) before conducting western blot analysis using another mAb (Abcam) in order to maximize detection specificity. Surprisingly, frataxin from erythrocytes (Fig. 1c, lane 2) showed a major band that migrated slightly slower than the mature frataxin from platelets (Fig. 1c, lane 3) and HepG2 cells (Fig. 1c, lane 4). Interestingly, frataxin from the whole blood lysates showed two bands (Fig. 1c, lane 1), aligning with the erythrocyte form (Fig. 1c, lane 2) and mature mitochondrial form of frataxin (Fig. 1c, lane 3,4). A more rigorous preparation of erythrocytes, which included several washes using PBS, eliminated the lower band corresponding to mature frataxin (Fig. 1c, lane 2). Previous studies showed an unusual migration of frataxin isoforms on glycine-SDS gels so this new isoform could conceivably have arisen through a truncation or a post-translational modification (PTM) of mature frataxin^23^. To clearly show that the erythrocyte frataxin and the mature form frataxin are two independent forms, we ran erythrocyte lysate with frataxin SILAC standard that showed only one band (Fig. 1d, lane 2) with higher molecular weight than the one corresponding to the band coming from the HepG2 lysate with frataxin SILAC standard (Fig. 1d, lane 3). To eliminate the possibility that within the erythrocyte milieu the mature frataxin form could be modified to a different isoform, we mixed the erythrocyte lysate with the HepG2 lysate and added the frataxin SILAC standard (Fig. 1d, lane 4). The subsequent western blot showed three clearly resolved bands from the mixture in which the mature form and SILAC standard^22^ were unaltered and were separated from the erythrocyte isoform (Fig. 1d, lane 4). This confirmed that a unique form of frataxin exists in erythrocytes, which we have named frataxin isoform E.

### Frataxin isoform E is extended from the N-terminus compared to the mature form

**Figure 2:**
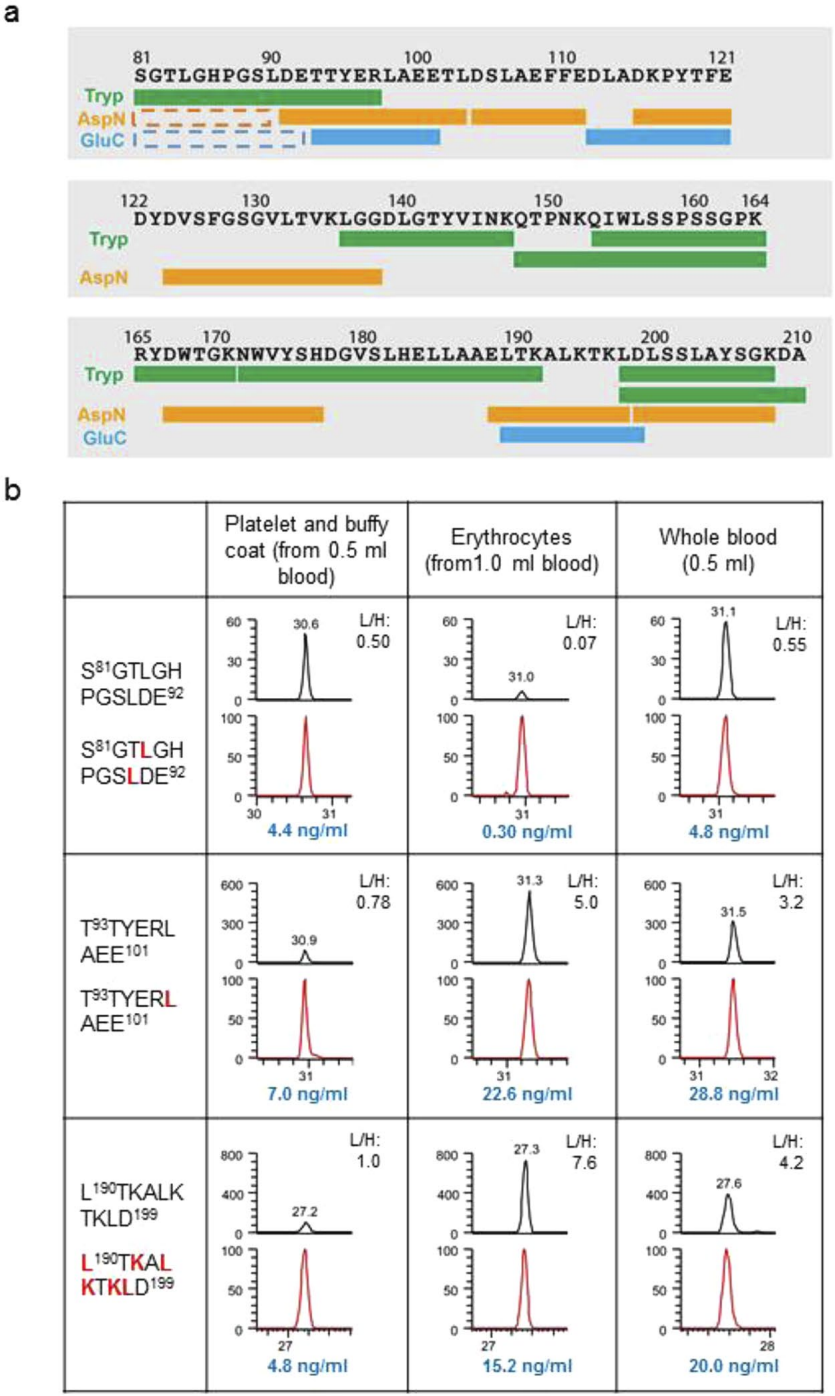
Erythrocyte frataxin is longer than the mature form at the N-terminus. (**a**) Diagram showing the peptides without PTMs detected in the frataxin by LC-MS. Different colors represent the enzymes used for digestion - trypsin (Tryp, green), AspN (orange), GluC (blue). The dashed line boxes represent the AspN and GluC peptides that were present in the standard mature frataxin protein digestion but were at very low levels from the erythrocyte lysate. (**b**) LC-MS chromatograms of GluC generated peptides from frataxin in different blood fractions. Black lines: endogenous frataxin; Red lines: SILAC frataxin standard. The heavy labeled amino acids in each peptide are shown in red. Y-axis: relative abundance to the heavy SILAC peptide signals. X-axis: time (min). Ratios of the light versus heavy peptide peak areas based on three or four MS transitions (Supplementary Table 1) were shown in each cell. The blue text shows the calculated frataxin protein amounts containing the indicated peptides.

The slow migration of isoform E suggests it might have a different sequence and/or have a PTM. We previously described a method for quantifying frataxin from platelets using IP, enzyme digestion and liquid chromatography-mass spectrometry (LC-MS)^22^. By combining LC-MS analysis of peptides from trypsin, AspN and GluC digestion, we showed that 98.5% of the mature form frataxin sequence was present in isoform E, and that no PTMs were present (Fig. 2a). Interestingly, although the platelet and standard mature frataxin generated S^81^GTLGHPGSL^90^ peptide from the AspN digestion, and S^81^GTLGHPGSLDE^92^ peptide from the GluC digestion, they were largely missing from the erythrocyte preparation (Fig. 2a,b; Supplementary Fig. 1). To further illustrate this point, we fractionated the platelets, leukocyte-enriched buffy coat and erythrocytes from whole blood. Peptides from the GluC digestion were used to calculate the frataxin levels in individual fractions. The calculation based on the second GluC peptide from the N-terminus T^93^TYERLAEE^101^ and a peptide close to the C-terminus L^190^TKALKTKLD^199^ resulted in consistent protein levels in all fractions: 4.8–7.0 ng/mL from platelets and buffy coat, respectively and 15.2–22.6 ng/mL from the erythrocytes, with the sum of the two close to the levels in the whole blood (20.0–28.8 ng/mL). However, although the calculation from the first peptide S^81^GTLGHPGSLDE^92^ yielded a consistent frataxin amount in the platelets and buffy coat (4.4 ng/mL) compared to the calculation based on the other two peptides, its level in the erythrocyte fraction was only 0.30 ng/mL, or approximately 1.3–2.0% of the erythrocyte frataxin amounts determined by the other two peptides. Interestingly, in the whole blood, the frataxin containing the S^81^GTLGHPGSLDE^92^ peptide was only 4.8 ng/mL, suggesting it was almost exclusively from the platelets and leukocytes. These results suggest that the erythrocyte frataxin is extended from the N-terminus of the mature frataxin sequence, with lysine or arginine prior to the S81 residue, which is susceptible to trypsin digestion.

### Erythrocyte frataxin contains amino acids 76–210

**Figure 3:**
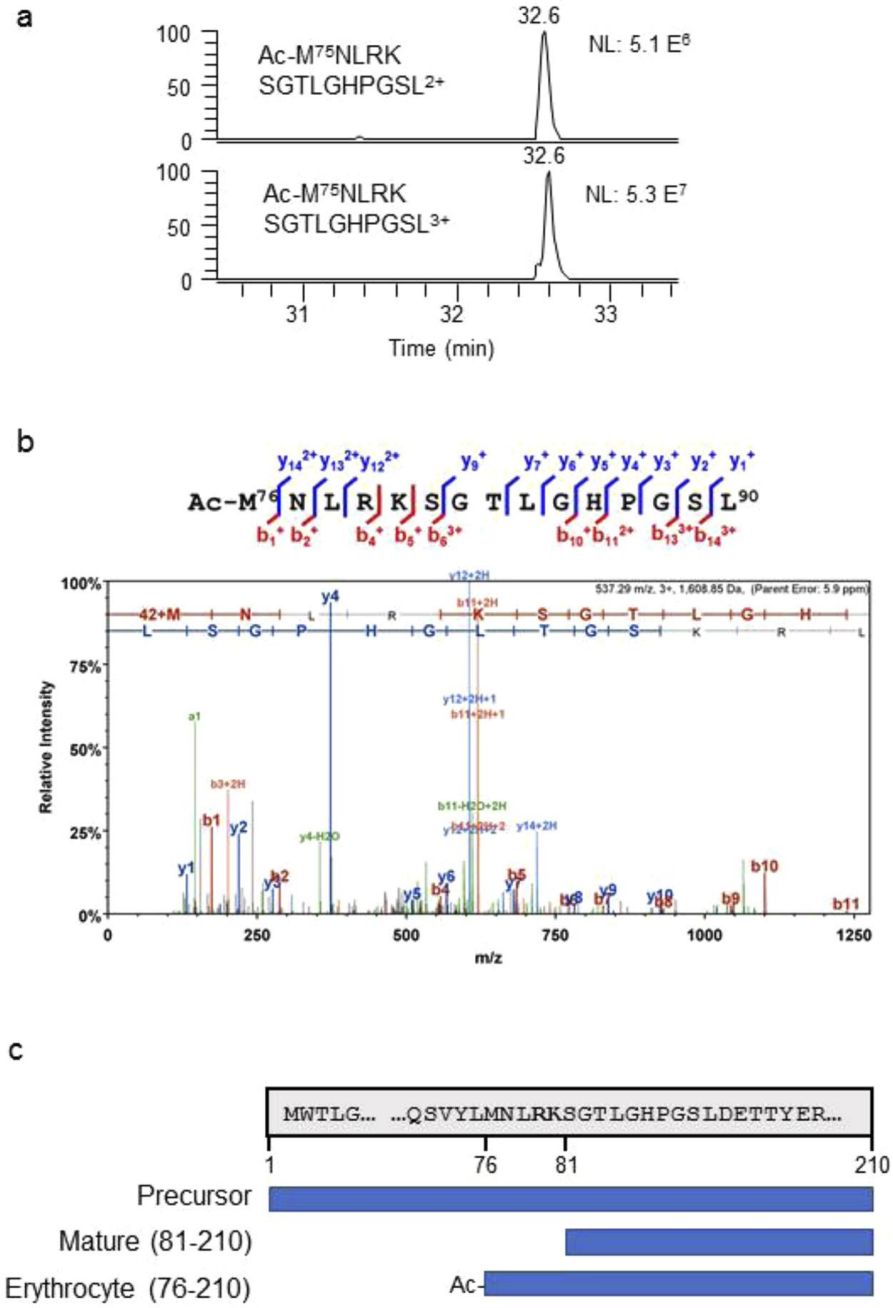
LC-MS analysis revealed the N-terminal sequence of erythrocyte frataxin. (**a**) Chromatograms of the unique N-terminal AspN peptide from erythrocyte frataxin. Top panel: doubly charged ion of the intact peptide (MH_2_^2+^); bottom panel: triply charged ion of the intact peptide (MH_3_^3+^). The MS signal intensities are shown on the right (5.1 × 10^6^, 5.3 × 10^7^). (**b**) Product ion spectrum of the N-terminal peptide of frataxin isoform E obtained from erythrocytes by AspN digestion. The b- and y-ions are labeled on the peptide and spectrum. (**c**) A diagram showing the amino acids present in the full length (precursor) frataxin, mature form (81–210) and erythrocyte frataxin (isoform E, 76–201).

We searched for erythrocyte frataxin unique peptides in the AspN and GluC digested samples. From AspN digested samples, we discovered a high abundance peptide, which had the following sequence: M^76^NLRKSGTLGHPGSL^90^. The peptide was exclusively acetylated at the N-terminus (Fig. 3a,b; Supplementary Fig. 2, Table 2), and the non-acetylated form was not detectable. The triply charged ion has much stronger signal than the double charged one (Fig. 3a). Interestingly, the sequence of this peptide aligns with the full-length frataxin isoform A amino acids 76–90. We synthesized Acetyl-MNLRKSGTLGHPGSL with three [^13^C_6_^15^N_1_]-leucine residues and found that it co-eluted with the endogenous erythrocyte peptide, which further confirmed its sequence (Supplementary Fig. 3). Therefore, isoform E starts from M^76^, which is five amino acids longer than the mature form (Fig. 3c).

### Erythrocyte frataxin is a novel isoform of frataxin

**Figure 4:**
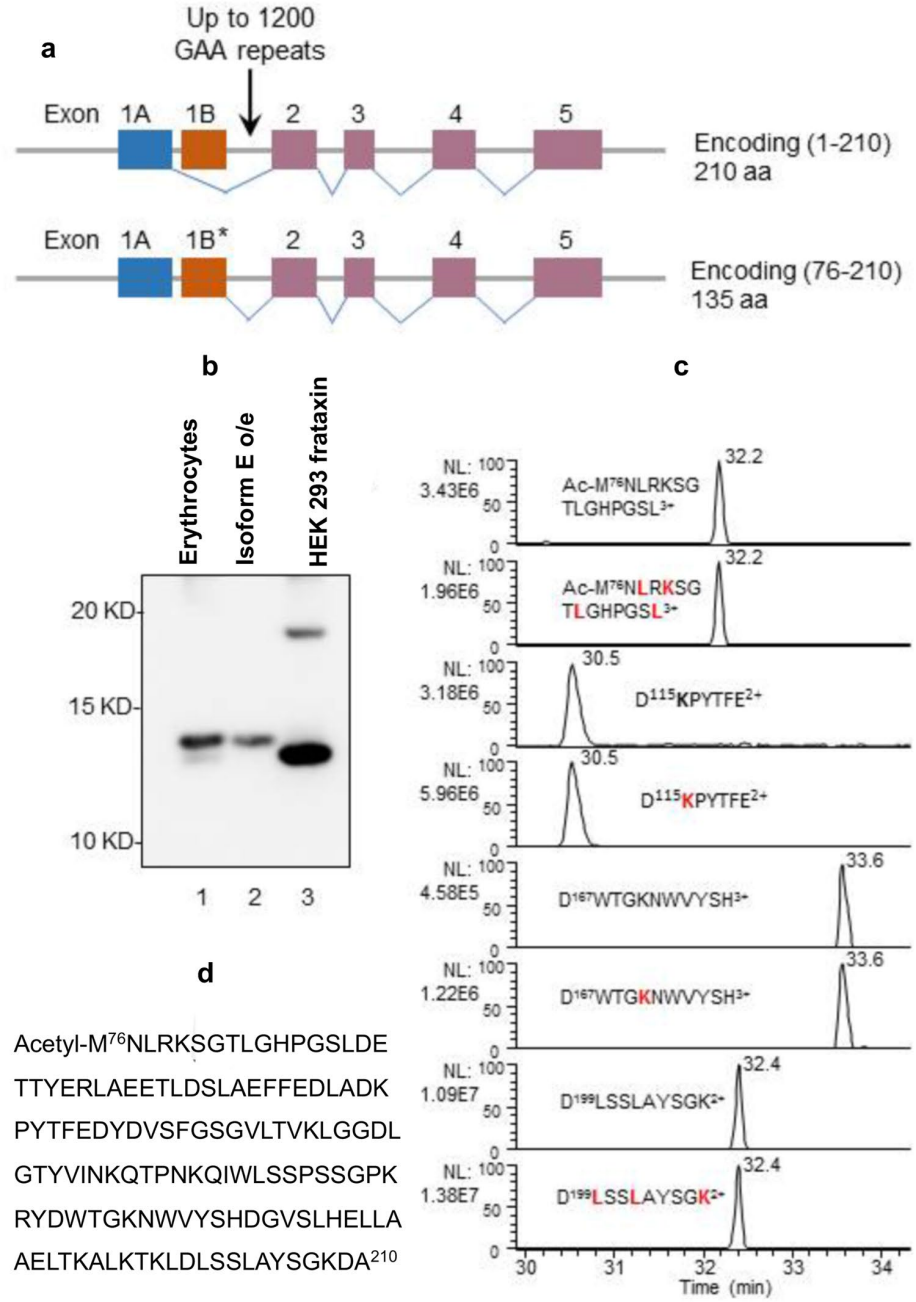
Erythrocyte frataxin is a novel isoform. (**a**) Diagram showing the mRNA that generates the canonical full length frataxin isoform A (1–210) and the alternative splicing of frataxin mRNA that generates isoform E (76–210). The first 18 nucleotides were missing in some transcripts. The figure is a modification of that published by Xia *et al*.^14^. (**b**) Western blot analysis of erythrocyte frataxin (lane 1), recombinant isoform E overexpressed (o/e) in HEK 293 cells (lane 2), and HEK 293 cell frataxin (lane 3). For endogenous frataxin detection, 10-fold more total protein was loaded in lane 3 compared to that in lane 2. (**c**) LC-MS chromatograms of representative AspN digestion peptides from a mixture of erythrocyte frataxin and isoform E overexpressed in SILAC HEK 293 cells labeled with stable isotope leucine and lysine, which are shown in red. (**d**) The complete sequence of frataxin isoform E (76–210) showing that it contains 135 amino acids.

The acetylation of methionine at the N-terminus occurs co-translationally^24^. Xia *et al*., previously discovered various *FXN* transcripts that were different from the canonical transcript, which generates full length frataxin with a mitochondrial targeting sequence^14^. One of the transcripts contained a novel exon 1B or an exon 1B missing the first 18 nucleotides (Fig. 4a). This transcript lacks the canonical start codon and the theoretical protein product starts with the subsequent ATG which corresponds to M^76^. We cloned the open reading frame of this transcript and the expressed protein co-migrated with isoform E on SDS-PAGE (Fig. 4b). SILAC isoform E was also expressed in HEK 293 cells and the cell lysate mixed with erythrocyte lysate. Frataxin IP was then conducted followed by AspN digestion and LC-MS analysis. The AspN-generated unlabeled frataxin peptides from erythrocyte frataxin were found to co-elute with the labeled peptides from the SILAC-isoform E that had been over-expressed in HEK 293 cells (Fig. 4c). This unequivocally confirmed the 135-amino acid sequence of isoform E shown in Fig. 4d.

### Erythrocytes are a major source of frataxin in whole blood

**Figure 5:**
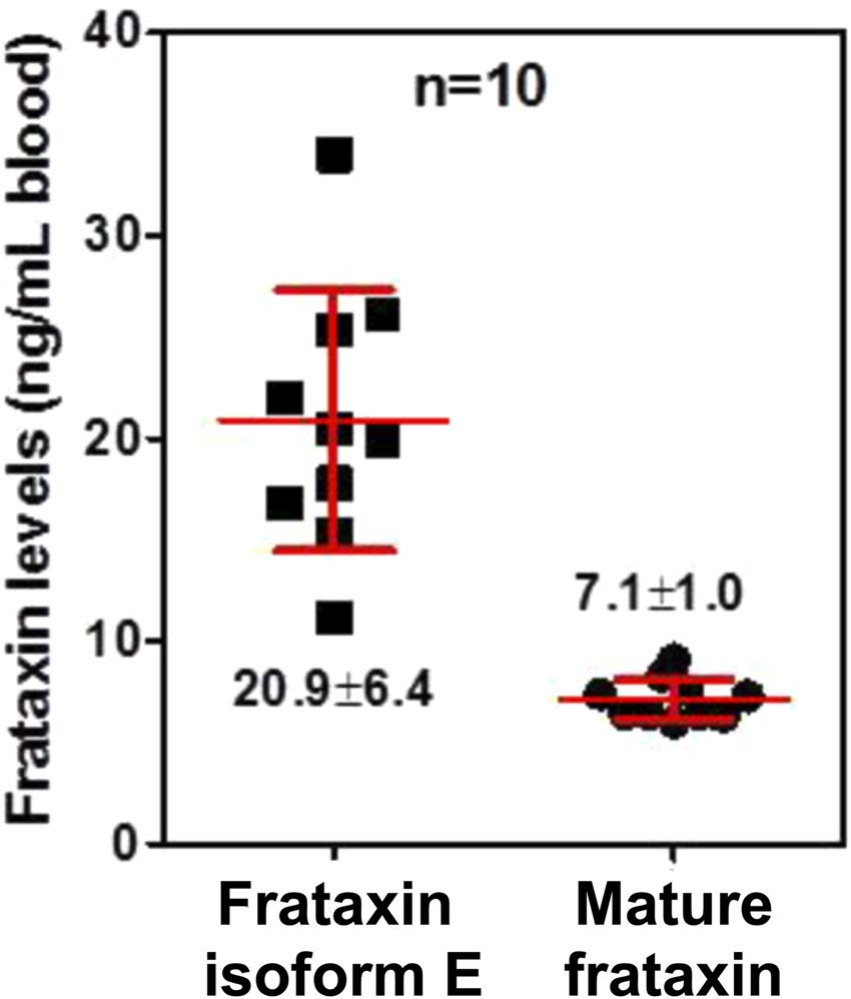
Levels of mature frataxin and isoform E in whole blood. Mature frataxin and isoform E levels in whole blood from healthy donors with GAA repeats < 40 were determined by stable isotope dilution LC-MS. Shown are the mean value ± standard deviation (n = 10). The variability in the amount of frataxin isoform E in erythrocytes compared to mature frataxin in cells with mitochondria probably arises from its different mechanism of formation through alternative splicing and N-terminal acetylation during translation rather than the normal transcription and translation involved in expression of full-length frataxin.

The differences in N-terminal sequence between isoform E and mature frataxin enabled us to distinguish them by LC-MS. We monitored the N-terminal peptides that are unique to the individual forms, together with three common peptides generated by AspN digestion of frataxin in whole blood from ten healthy donors with two normal *FXN* alleles (GAA repeat lengths <40). Quantification of S^81^GTLGHPGSL^92^ using stable isotope dilution LC-MS methodology used previously for tryptic peptides^22^, showed that there was 7.1 ± 1.0 ng/mL of mature mitochondrial frataxin (81–210) in the whole blood (Fig. 5). Direct quantification of the Ac-M^76^NLRKSGTLGHPGSL^92^ peptide is not very accurate because differential methionine oxidation occurs after sample isolation and digestion. Therefore, we calculated the amount of isoform E by subtracting the amount of mature mitochondrial frataxin from the total frataxin. This revealed a 3-fold higher level of isoform E in the whole blood (20.9 ± 6.4 ng/mL) compared with mature frataxin (Fig. 5). One round of freeze-thawing had no effect on the concentrations of either isoform E or mature frataxin. Semi-quantitative analysis using the heavy isotope labeled peptide Ac-M^76^N[^13^C_6_^15^N_1_]LKSGT[^13^C_6_^15^N_1_]LGHPGS[^13^C_6_^15^N_1_]L^92^ as the internal standard (added after trypsin digestion) found similar levels of isoform E.

### Frataxin isoform E is localized in cellular cytosol and nucleus

**Figure 6:**
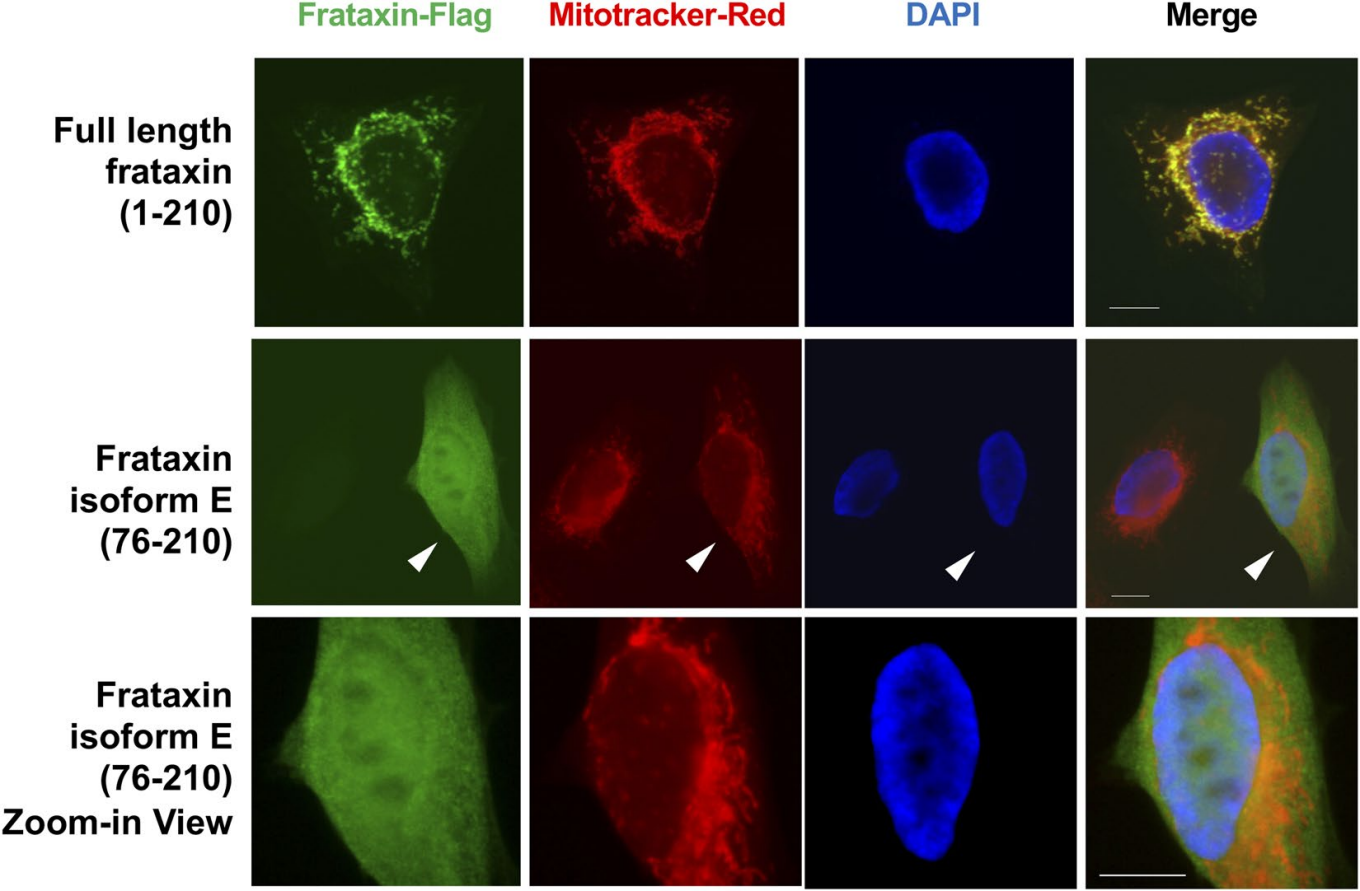
Frataxin isoform E is distributed in the cytoplasm and nucleus. HeLa cells expressing Flag-tag frataxin full length or isoform E were stained with an anti-Flag antibody. The mitochondria are shown by MitoTracker Red. Nuclei were visualized by DAPI staining. The middle panel shows (with an arrowhead) a cell expressing frataxin isoform E (76–210). The bottom panel shows a zoom-in view of the cell indicated by the arrowhead in the middle panel. The scale bar represents 10 µm.

Erythrocytes do not have mitochondria and so frataxin is located in the cytoplasm. Consistent with this notion, isoform E lacks the mitochondrial targeting sequence located at the N-terminus of full length frataxin isoform A^25^. To identify the localization of isoform E in normal cells with nuclei and mitochondria, we expressed a C-terminal Flag-tag form in HeLa cells as well as a Flag-tag form of the canonical full-length frataxin isoform A. Anti-Flag detection showed that canonical full length frataxin isoform A co-localized with the mitochondria that were visualized by MitoTracker Red (Fig. 6, upper). In contrast, isoform E displayed a diffuse pattern in both cytosol and nucleus with no enrichment in the mitochondria (Fig. 6, middle, right cell shown by an arrowhead). Isoform E was excluded from many DAPI-negative regions which represent nucleoli (Fig. 6, lower). As a control, no fluorescence signal was detected in an adjacent non-transfected cell (Fig. 6, middle, left cell).

### Frataxin isoform E can be cleaved into mature frataxin (81–210) in cells

**Figure 7:**
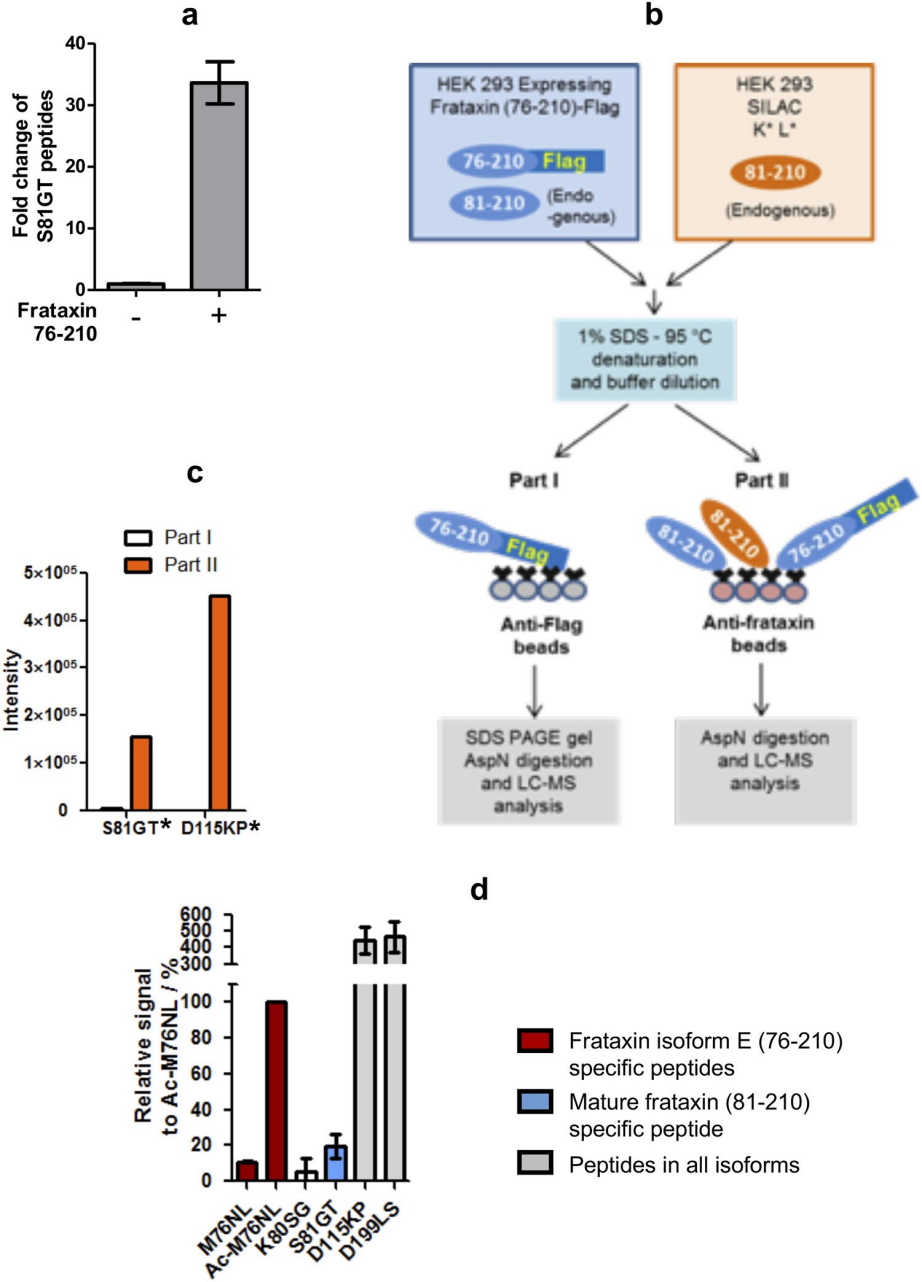
Expression of frataxin isoform E leads to the formation of mature frataxin in cells. (**a**) Frataxin 76–210 was overexpressed in HEK 293 cells. The whole cell lysates were analyzed by LC-MS after IP and AspN digestion. Levels of frataxin containing the mature form specific peptide (SGTLGHPGSLED) were determined. (**b**) A diagram showing the LC-MS analysis of Flag-tag frataxin 76–210. See Methods section for detailed experimental procedure. (**c**) LC-MS signal intensities of representative heavy isotope-labeled peptides from AspN digestion of samples prepared as in (**b**). The asterisks indicate that peptides contained the stable isotope labeled K and L amino acids shown in panel (**b**). (**d**) Relative LC-MS signal intensities of peptides from AspN digestion of frataxin 76–210 Flag IP (as shown in b, part I). Cells were harvested at 24, 48 and 72 h after transfection. As no major difference was observed among these samples, the peptide signal intensities were normalized to the LC-MS response of the first peptide of isoform E (Ac-MNLRKSGTLGHPGSLED, Ac-M76N) and the average of relative signals ± standard deviation are shown. The peptides from all isoforms (D115KP, D199LS, (**d**) had significantly more intensity than the N-terminal peptides because of their better ionization efficiency. However, the ratio of their intensities to the sum of the amino acid terminal peptide intensities (M76NL, AcM76NL, K80SG, S81SG, (**d**) was similar to that observed in mature frataxin or isoform E. See Supplementary Table 1 for detailed peptide information.

Transcripts that could yield isoform E (named frataxin II) were identified in HEK 293 cells and human muscles by Xia *et al*.^14^. However, there are no reports describing detection of the corresponding protein in the cytosol of cells. In contrast, there are several studies showing the presence of mature mitochondrial frataxin in the cytosol^20,26–30^. LC-MS analysis revealed only a trace amount of isoform E in HEK 293 cells, corresponding to approximately 2–3% of the mature mitochondrial form. In order to investigate the stability of frataxin isoform E in cells other than erythrocytes, an untagged construct was over-expressed in HEK 293 cells. This resulted in a 30-fold increase in the concentration of mature mitochondrial frataxin (81–210) (Fig. 7a). The increase in mature frataxin could have resulted either from proteolytic cleavage of isoform E or from an indirect effect on processing of endogenous full-length frataxin A. To address these issues, C-terminal Flag-tag isoform E was expressed in HEK 293 cells, and the resulting frataxin isoforms were separated from endogenous frataxin by IP using anti-Flag M2 beads. Frataxin can form oligomers in cells^31,32^ and so a denaturing IP procedure was performed to prevent binding of endogenous frataxin to the Flag-tag protein. The separation efficiency was monitored by adding equal amounts of SILAC HEK 293 cell lysate protein into the unlabeled lysates containing isoform E-Flag (Fig. 7b). No isotope-labeled frataxin peptides were detected in the anti-Flag IP peptides showing that only Flag-tag peptides were isolated (Fig. 7c Part I). In contrast, when the cell lysate mixture was IPed by anti-frataxin beads (rather than the anti-Flag beads), strong LC-MS signals were observed for the labeled peptides from mature frataxin (Fig. 7 Part II). Importantly, the anti-Flag IP yielded intense LC-MS signals from the mature form-specific AspN peptide S^81^GTLGHPGSL^90^. The signal intensity was approximately 20% of the isoform E-specific AspN peptide Ac-M^76^NLRKSGTLGHPGSL^90^ (S^81^GT Fig. 7d). Minor amounts of non-acetylated isoform E (M76NL, Fig. 7d) and a truncated form of mature frataxin where cleavage had occurred between R-79 and K-80 (K80SG, Fig. 7d) were observed. Two peptides common to all isoforms (D115KP, D199LS, Fig. 7d) were significantly more intense because of their better ionization efficiency. However, the ratio of their intensities to the sum of the amino acid terminal peptide intensities (M76NL, AcM76NL, K80SG, S81SG, Fig. 7d) was similar to those observed in mature frataxin or isoform E.

## Discussion

The structure and function of frataxin has been the focus of a substantial amount of research since it was discovered as a mediator of iron homeostasis^33^, and a factor required for the assembly of mammalian iron-sulfur cluster proteins^34^. Furthermore, the discovery that decreased expression of frataxin causes the neurodegenerative and cardiodegenerative disease of FA^35^ has provided a separate impetus for additional functional studies. Mature biologically active frataxin arises through mitochondrial processing of full length frataxin isoform A^11,23^ and its reduced mitochondrial expression is responsible for the adverse effects observed in FA^8^. Previous studies have suggested that mitochondrial frataxin catalyzes the ferrochelatase-mediated conversion of protoporphyrin IX to heme, as the final step of heme biosynthesis^18,36–40^. However, these findings are somewhat controversial as there are conflicting reports on how frataxin affects heme biosynthesis. In human lymphoblasts, frataxin deficiency was found to cause a decrease in the biosynthesis of heme metabolites^19^; whereas, in erythroid progenitor cells no changes in heme synthesis were observed^21^. The presence of mature frataxin in erythrocytes, a cell that lacks mitochondria, is difficult to reconcile with this paradigm.

Several previous studies have proposed a role for extra-mitochondrial frataxin in human^20,26–30^ but not mouse^41^ iron-sulfur cluster protein formation. The lack of mitochondria in erythrocytes suggested that they could contain an extra-mitochondrial form of frataxin that is different from the mature form in mitochondria. A combination of high sensitivity LC-MS coupled with molecular biology techniques has now unequivocally confirmed the presence of an alternatively spliced form of frataxin in erythrocytes. Translation of this novel protein starts from the second methionine (M^76^) of the canonical form (isoform A) rather than the first methionine (M^1^). Erythrocyte frataxin is also acetylated at the N-terminus during translation to give isoform E. N-terminal acetylation would be predicted because when the bulky amino acid asparagine is adjacent to the N-terminal methionine residue, methionine aminopeptidase is no longer active during translation. Instead, N-acetyl transferase B catalyzes the transfer of an acetyl group from acetyl-coenzyme A to the N-terminal methionine residue^24^.

**Figure 8:**
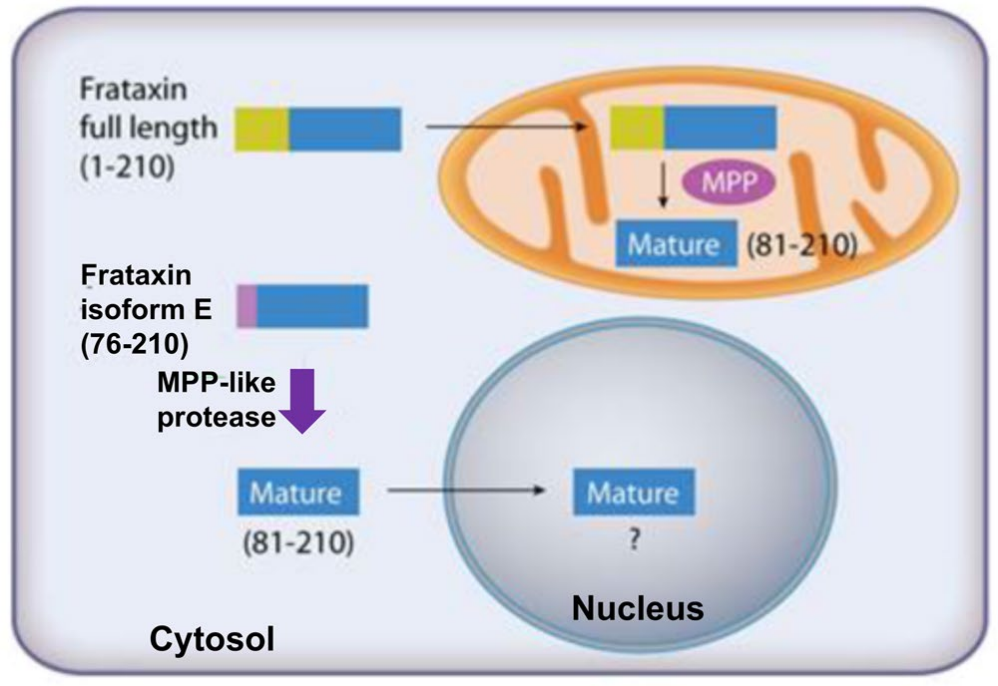
The role of frataxin isoforms in cells. Canonical full-length frataxin isoform A (1–210) is targeted to mitochondria, where the first 80 amino acids (including the mitochondrial targeting sequence) are removed by MPP to give the mature form (81–210). Frataxin isoform E (76–210) does not contain a mitochondrial targeting sequence, and so it is retained in the cytosol where it is cleaved by an MPP-like protease to give extra-mitochondrial mature frataxin identical with that found in the mitochondria. It has been suggested by Lill *et al*. that extra-mitochondrial frataxin could facilitate the assembly of iron sulfur cluster proteins involved in DNA repair and telomere length control^4^. Erythrocytes, which lack mitochondria, contain only isoform E. They also lack the MPP-like protease present in the cytosol of non-hematopoietic cells.

Alternative splicing to generate protein isoforms targeted to different cellular compartments has been reported previously for human NFU1, another protein involved iron-sulfur cluster formation^42^. Interestingly, it is possible to detect alternatively spliced isoform E at a level of 2–3% of mature form frataxin in HEK 293 cells. Therefore, we speculated that isoform E could be processed into a truncated form in the cytosol of non-erythroid cells leaving only a residual amount of the full-length protein. This possibility is confirmed by over-expressing Flag-tag isoform E in cells containing nuclei and mitochondria and detection of shorter Flag-tag form of frataxin. The shorter frataxin isoform arises primarily through proteolytic cleavage at K^80^, leading to a sequence identical with the mitochondrial mature form (Fig. 8). Proteolysis also occurs at R^79^, although at a much lower rate (Fig. 7d) so the enzyme has a similar specificity to MPP^10^ but is a little more promiscuous. A previous report described expression of a potential frataxin degradation product frataxin (78–210)^23^. It is possible to detect this isoform in HEK 293 cells over-expressing isoform E, but it is present at very low levels. Previous studies have shown that frataxin 81–210 migrates slower on Tris-Glycine gels than frataxin (79–210) and frataxin (76–210)^11,23^. In contrast, under conditions used in the present study, isoform E and mature frataxin (81–210) migrate at their predicted molecular weights. This is most likely because bis-Tris gels were used with MES running buffer instead of the MOPS buffer used previously.

Expression of isoform E with a Flag-tag also reveals that it has both cytosolic and nuclear localization (Fig. 6, lower). This suggests a possible role for extra-mitochondrial frataxin in both cellular compartments. Previous studies have reported the existence of endogenous frataxin in human cell cytosol fractions^20,26–30^. For example, Condo *et al*. showed that over-expressed frataxin 81–210 interacts with cytosolic aconitase/iron regulatory protein-1 (IRP1)^30^. In a similar manner to mitochondrial frataxin and aconitase/IRP1 interaction, extra-mitochondrial frataxin facilitates the cytosolic switch of this bifunctional protein between an enzymatic and a RNA-binding function. In addition to a cytosolic function, there are several studies showing that enhanced DNA damage^43,44^ and telomere shortening^45^ occurs in frataxin deficient animals and FA patients. The increased DNA damage in microglial cells with frataxin deficiency may contribute to the neurodegeneration observed in FA^46^, as many iron-sulfur cluster (ISC) proteins are involved in DNA repair and telomere stability. These studies suggest a potential function of frataxin in the nucleus (Fig. 8). Interestingly, both the endogenous cytosolic and nuclear frataxin identified in these studies appear similar in size to the mature mitochondrial form based on SDS-PAGE analysis^30,47^. It seems likely that the mature form of frataxin is derived from protease cleavage of a longer extra-mitochondrial isoform, such as isoform E (Fig. 8).

In order to analyze erythrocyte frataxin protein by LC-MS, we first selectively lysed the erythrocytes and removed the intact platelets and leukocytes, which contain >90% of the mitochondria in whole blood cells^22^. Almost 98% of the erythrocyte frataxin is present as the alternatively spliced isoform E with <2% contamination by the mature mitochondrial form of frataxin (81–210) (Fig. 2b). Because blood contains 0.5–1.5% reticulocytes, they may be the source of the residual mature mitochondrial frataxin. Many cytosolic and membrane proteins found in erythrocytes are expressed in pre-reticulocytes such as erythroblasts, which have a nucleus, and in reticulocytes that retain their ribosomal machinery and mitochondria^48–50^. Such proteins are resistant to degradation during erythropoiesis to non-proliferating erythrocytes when the nucleus, endoplasmic reticulum, ribosomes, and mitochondria are shed^51^.

LC-MS analysis has previously identified >2,650 proteins in erythrocytes including >1100 non-membrane cytosolic proteins^48–50^. Frataxin isoform E, which was not detected in these previous LC-MS studies, appears to be another cytosolic protein that has survived during erythropoiesis. Surprisingly, it was not detected as one of the 287 N-terminally acetylated proteins identified by polymer-based depletion of internal and C-terminal proteolytic digests of the erythrocyte proteome^52^. This highlights the power of combining a stable isotope analog as a carrier for low abundance proteins when IP procedures are employed in combination with LC-MS methods^53^. Due to the minor molecular weight difference between the mature form and frataxin isoform E, it is challenging to analyze the new isoform and its cleavage products by SDS-PAGE and immunoaffinity-based assays. In contrast, by using AspN digestion, we can identify N-terminal peptides specific to the mature form or isoform E (Fig. 3). This provides a significant advantage because it is then possible to use LC-MS to distinguish the two forms in blood cells.

Frataxin isoform E could be involved in the assembly of extra-mitochondrial iron-sulfur cluster proteins in a similar manner to the slightly shorter mitochondrial form found in mitochondria^54^. N-terminal acetylation of isoform E is particularly relevant because N-terminal domains are important in iron-sulfur cluster formation^55^. Three other proteins that are also involved in iron-sulfur clusters have previously been identified in erythrocytes – CIAO1, CIAPIN1, and CISD2^49,50^. CIAO1 (cytosolic iron-sulfur protein assembly-1) associates with either cytosolic iron-sulfur cluster assembly (CIA) 2A or CIA2B proteins and the CIA-targeting factor MMS19 in assembly of the iron regulatory protein 1 (IRP1) that is critical for cellular iron homeostasis^30^. IRP-1, which is cytosolic aconitase lacking iron^30^, was not detected by LC-MS^48–50,52^, although aconitase has been identified previously at low levels in erythrocytes by functional assays^56^. Normal adults have approximately 4 g of iron in their body with some 75% of the iron found in erythrocyte hemoglobin. This means that the control of iron homeostasis and the maintenance of iron-sulfur cluster proteins are likely important functions of frataxin isoform E during erythropoiesis. CIAPIN1 (cytokine-induced anti-apoptosis inhibitor-1, anamorsin) is involved in cytosolic Fe-S cluster assembly and distribution to numerous proteins. It was also recently implicated in the mitoNEET (NSD1)-mediated recycling of cytosolic apo-IRP1 into holo-aconitase^57^. Finally, the NEET proteins (CISD1 and CISD2) belong to a newly discovered class of iron-sulfur proteins required for the regulation of iron homeostasis^58^.

The presence of iron-sulfur cluster proteins in erythrocytes, means that frataxin isoform E could play a role in their assembly at the pre-erythrocyte stages of erythropoiesis. However, it is not obvious how such a role would be useful in mature erythrocytes because iron-sulfur cluster formation would have already taken place. It is noteworthy that frataxin mRNA from the naked mole rat (Heterocephalus glaber) codes for a 135 amino acid protein (NCBI protein: EHB01040.1; Uniprot *FXN*: G5AVH9) that has 90.3% similarity and 98.5% homology with human isoform E. Furthermore, frataxin mRNA from Brandt’s bats (Myotis brandtii) codes for a 135 amino acid protein (NCBI protein: XP_014396790.1) that has even greater similarity (91.9%) and homology (99.3%) with human isoform E. Naked mole rats^59^ and Brandt’s bats^60^ live five times and ten times longer, respectively than would be predicted from their body mass^61^. In contrast, rats (Uniprot *FXN*: M0RAK4) and mice (Uniprot *FXN*: O35943), mammals that do not express frataxin isoform E, live shorter lives than predicted (longevity factors 0.6 and 0.7, respectively)^61^ suggesting that isoform E could play an unrecognized biochemical role in the prevention of aging by facilitating DNA repair and/or telomere length control^61,62^.

Lazaropoulos *et al*.^17^ identified two individuals who carry truncation mutations in the *FXN* gene (c.2delT and c.11–12delTC) that code for the first 5 amino acids in frataxin protein. These individuals had blood frataxin levels that were in the expected range for carriers or healthy controls, while having buccal cell frataxin levels in the lower portion of the patient range. A third subject who carried a c.2delT mutation in conjunction with a pathologic but short (90) GAA repeat had disease levels in buccal cell (22%) with high control levels (157%) in blood. The distribution of frataxin in blood was examined in more detail in one subject with the c.2delT mutation (subject P.A. 002 in Deutsch *et al*.^62^). The red blood cell pellet, which had the highest absolute amounts of frataxin, was responsible for the relatively elevated frataxin level seen in whole blood. It was suggested that these truncation mutations in *FXN* retained immunoreactive frataxin in erythrocytes through an unknown mechanism^17^. Our finding that isoform E is a splice variant of frataxin that would not be affected by the c.2delT and c.11-12delTC mutations now provides an explanation for these previously puzzling observations.

Due to the long half-life of erythrocytes (115 days)^63^, its frataxin level will not reflect immediate frataxin level changes upon drug treatments. Therefore, previous analysis using frataxin in platelets and PBMCs as biomarkers have relied on cell fractionation from freshly collected whole blood^22^. The finding that erythrocyte frataxin has a different molecular form will now make it possible to directly utilize whole blood for frataxin analyses, significantly reducing the sample volumes and assay complexity, as well as making it possible to analyze mature frataxin levels in frozen whole blood samples. Furthermore, the ability to specifically quantify the different molecular forms in erythrocytes and other blood cells will be extremely useful for monitoring the natural history of diseases such as FA. Finally, our finding of an extra-mitochondrial form of frataxin raises the possibility that FA might not be simply a mitochondrial disease and that decreased extra-mitochondrial frataxin could contribute to disease etiology (Fig. 8). Monitoring the natural history of those FA patients that can express normal extra-mitochondrial frataxin with reduced levels of mitochondrial frataxin^17^ will provide further insight into this possibility.

## Methods

### Reagents

All reagents and solvents were LC-MS grade quality unless otherwise noted. [^13^C_6_^15^N_2_]-lysine and [^13^C_6_^15^N_1_]-leucine were from Cambridge Isotope Laboratories (Andover, MA, USA). Anti-frataxin mouse mAb (clone 1D9) for cross-linking to magnetic beads was from LifeSpan Biosciences, Inc. (Seattle, WA), and the anti-frataxin mouse mAb for western blot analysis was 17A11 from Abcam (Cambridge, MA). LifeSpan Biosciences has discontinued clone 1D9. However, Abcam anti-frataxin rabbit polyclonal Ab175402 or mouse monoclonal Ab113691 can be used as alternatives in the IP procedure. EDTA-free protease inhibitor cocktail, DL-dithiothreitol (DTT), dimethyl pimelimidate dihydrochloride (DMP) were purchased from MilliporeSigma (Billerica, MA). LC grade water and acetonitrile were from Burdick and Jackson (Muskegon, MI, USA). Protein G magnetic beads were obtained from Life Technologies Corporation (Grand Island, NY). The heavy isotope leucine labeled AQUA peptide (Acetyl-MNL*RKSGTL*GHPGSL* with L* = [^13^C_6_^15^N_1_]-L) was synthesized by Thermo Scientific (isotopic enrichment > 99%, HeavyPeptide AQUA custom synthesis service (Rockford, IL, USA).

### Clinical samples

Blood samples were obtained from healthy donors (GAA repeat lengths <40) enrolled in an ongoing natural history study of FA in accordance with the Declaration of Helsinki and the approved guidelines of the Children’s Hospital of Philadelphia. Written informed consent was obtained from each donor participating in the study. If subjects were under the age of 18, written informed consent was obtained from a parent and/or legal guardian. The study was approved by the Institutional Review Board (IRB) of the Children’s Hospital of Philadelphia (IRB Protocol # 01-002609).

### Erythrocyte, platelet, and buffy coat isolation of frataxin for IP

Venous blood was drawn into 8.5 mL acid-citrate-dextrose Vacutainer tubes. 2 mL of whole blood was saved as 0.5 mL aliquots for analysis. 4 mL of blood was transferred to 15-mL polypropylene tubes and spun at 200 g for 10 min at room temperature with no brakes. The upper platelet-rich layer and buffy coat layer were transferred to a 2 mL tube and spun at 800 g for 20 min. The plasma was removed, and the platelet and leukocyte pellets were carefully washed with 0.5 mL platelet wash buffer (10 mM sodium citrate, 150 mM NaCl, 1 mM EDTA, 1% (w/v) glucose, pH 7.4) by spinning at 800 g for 5 min. The erythrocyte fraction was washed once by mixing with equal volume of PBS and spun at 800 g for 20 min. The supernatant was removed, and erythrocytes were aliquoted in clean tubes. To eliminate contamination from platelets and leukocytes, erythrocyte lysates were also prepared. Erythrocytes from 1 mL whole blood were mixed with 14 mL ice-cold erythrocyte lysis buffer (8.02 g/L NH_4_Cl, 0.84 g/L NaHCO_3_ and 0.37 g/L disodium EDTA) in a 15 mL tube. The mixture was rocked for 15 min at room temperature until liquid is clear red. The samples were spun at 4 °C for 10 minutes at 500 g. The supernatant was transferred to an Amicon 3 kDa 15 mL filter and was concentrated to less than 1 mL by spinning at 4000 g at 4 °C and purified by IP.

### Detection of frataxin in different blood fractions

Platelet and buffy coat from 1 mL blood and whole blood, erythrocytes and plasma from 100 μL were dissolved in 300 μL IP-lysis buffer (50 mM Tris, pH 7.5, 150 mM NaCl, 0.5% Triton X-100, 0.5% NP-40, 1 mM DTT) supplemented with 1x complete protease cocktail and incubated on ice for 15 min. 50 µg platelet lysates, 70 µg buffy coat lysate and whole blood, erythrocyte and plasma lysates each from 40 μL whole blood were spiked with a lysine and leucine stable isotope labeled frataxin internal standard (1 ng)^22^. Samples were sonicated for 30 sec using a sonic dismembranator (Fisher) and centrifuged at 16,000 g using a bench top centrifuge for 15 min at 4 °C. The supernatant was applied to an Amicon Ultracel-50K filters (MilliporeSigma). The filters were spun at 4000 g, 4 °C for 20 min. The flow-through was transferred to Amicon Ultracel-3K filter units and spun at 4000 g, 4 °C for 40 min until the sample volume was concentrated to less than 50 μL. Samples were then subjected to western blot analysis and the results are shown in Fig. 1.

### SDS-PAGE and western blot

Cell lysate or eluted IP samples dissolved in LDS sample buffer were run on NuPAGE™ 12% bis-Tris protein gels (Invitrogen, Carlsbad, CA). NuPAGE™ MES SDS Buffer was used for optimal separation of proteins among 10–20 kDa. Frataxin was detected by mAb anti-Frataxin (17A11, Abcam, Cambridge, MA) and anti-mouse HRP (Santa Cruz Biotechnology, Dallas, TX). Western blots were developed using ECL reagents. Because mature frataxin is a protein of very low molecular weight (14.3 kDa), the membranes were cut at 25 or 50 kDa before application of antibodies. This protocol was used for the results shown in Fig. 1b.

### IP and digestion of frataxin

The protocol described previously^22^ was used with minor modifications. Briefly, anti-frataxin (clone 1D9, LifeSpan Biosciences, Seattle, WA) was cross-linked to protein G beads (Life Technologies Corporation, Grand Island, NY) through DMP as described previously^22^. Erythrocytes, platelets and buffy coat or whole blood were lysed in 4 mL ice-cold IP-lysis buffer (50 mM Tris, pH 7.5, 150 mM NaCl, 0.5% Triton X-100, 0.5% NP-40, 1 mM DTT) supplemented with 1x complete protease cocktail and incubated on ice for 15 min. A lysine and leucine stable isotope labeled frataxin internal standard (20 ng)^22^ was spiked in before pulse sonication for 30 sec using a sonic dismembranator (Fisher). Samples were centrifuged at 16,000 × g using a bench top centrifuge for 15 min at 4 °C. The supernatant was filtered using Amicon Ultracel-50K filters (MilliporeSigma, Burlington MA). The filters were spun at 4000xg, 4 °C for 20 min and the flow through was used for IP. The IP and protein elution procedure were exactely as described previously^22^. Briefly, samples were incubated with 0.3 mg anti-frataxin beads at 4 °C overnight and washed once with IP-lysis buffer and three times with PBS. For western blot analysis, the beads were boiled in 40 μL 1X NuPAGE LDS Sample Buffer (Invitrogen, Carlsbad, CA) at 95 °C for 5 min. For LC-MS analysis, frataxin was eluted by incubating the beads with 100 μL elution buffer (300 mM acetic acid and 10% acetonitrile) and dried under nitrogen flow. The samples were dissolved in 25 mM aqueous NH_4_HCO_3_ (50 μL) containing 50 ng AspN (MilliporeSigma, Billerica, MA) or 100 ng trypsin (Promega, Madison, WI). Alternatively, the samples were dissolved in 50 mM ammonium acetate pH 4 containing 1 μg Glu-C V8 Protease (MilliporeSigma, Billerica, MA). The samples were incubated at 37 °C overnight before LC-MS analysis as described below. This protocol was used to genernate the results shown in Figs 2b, 3a,b and 5.

### Constructs

Frataxin isoform II reported previously by Xia *et al*.^14^ was cloned into a pRK5 plasmid by PCR through BamHI and SalI restriction sites. The plasmid was a kind gift from Dr. Xiaolu Yang from University of Pennsylvania. Flag-tag frataxin full length or isoform II was cloned into pRK5 using the same restriction sites by including the FLAG sequence (translated as DYKDDDDK) immediately after the protein COOH-terminus sequence in the reverse primer. The template for PCR amplification of frataxin was purchased from Dharmacon, GE Life Sciences (gene accession number: BC048097).

### Cell culture and transfection

HEK 293 and HeLa cells (from ATCC) were maintained in DMEM supplemented with 10% fetal bovine serum, 100 units/mL penicillin, and 100 mg/liter streptomycin (all from Gibco laboratories, Gaithersburg, MD). For SILAC, HEK 293 cells were cultured in DMEM medium (Thermo Scientific) containing [^13^C_6_^15^N_2_]-lysine and [^13^C_6_^15^N_1_]-leucine for at least five passages. Plasmids were transfected into cells using Lipofectamine 2000 (Invitrogen, Carlsbad, CA) according to the manufacturer’s instruction. Transfection medium was replaced 6 h after initial transfection.

### Immunofluorescence of cultured cells

HeLa cells instead of HEK 293 cells were chosen for immunofluorescence study due to their stronger adherence on the coverslips. 24 h after transfection with frataxin full-length or frataxin isoform II fused to Flag-tag, HeLa cells were stained with cell culture medium containing 1 mM MitoTracker Red CMXRos (Invitrogen, Carlsbad, CA) for 30 min. Cells were washed twice with PBS and fixed with 4% paraformaldehyde for 15 min, permeabilized with 0.2% Triton X-100 for 15 min and blocked with 1% BSA for 30 min. Cells were stained with anti-Flag M2 antibody (1:400) (MilliporeSigma, Billerica, MA) and goat anti-mouse IgG Alexa Fluor 488 (Invitrogen, Carlsbad, CA). Cells were mounted with medium containing DAPI (for DNA detection) (Vector Labs), and the images were acquired with an RVL-100-B2 microscope (Echo Laboratories), and are shown in Fig. 6.

### Frataxin isoform E cleavage assay

HEK 293 cells were transfected with frataxin isoform-E-Flag. 24 h, 72 h and 48 h after the transfection, cells were harvested and lysed in 400 μL IP-lysis buffer. The protein concentrations of cell lysates were measured by Bradford assay (Bio-Rad, Hercules, CA) and each sample was mixed with SILAC HEK 293 cell lysate of the same protein amounts. 45 μL of 10% SDS was added to the lysate mixtures and samples were heated at 95 °C for 5 min. Samples were diluted by 4.5 mL ice-cold IP-lysis buffer. 2 mL of the samples was incubated with anti-frataxin coupled beads and 2 mL was incubated with anti-Flag M2 beads (MilliporeSigma) overnight on a rotator. Protein bound to the anti-frataxin beads were eluted and digested by AspN as described above. The anti-Flag bead-bound proteins were eluted by boiling in the LDS sample buffer and separated by SDS-PAGE to remove contaminating proteins that had also bound to the beads. The bands corresponding to the frataxin-Flag were cut and the gel slices were cut into 1 mm^3^. The gel pieces were washed twice with destain solution (25 mM NH_4_HCO_3_ in water/acetonitrile (1:1 v/v), once with acetonitrile, and incubated with 100 μL 25 mM NH4HCO_3_ containing 50 µg AspN. The samples were analyzed by LC-MS. This protocol was used for the results shown in Figs 4c and 7a,c,d.

### LC-MS analysis

The LC-MS analysis of peptides was performed as previously described^22^. Briefly, MS was conducted using a Q Exactive™ HF coupled to Dionex Ultimate 3000 RSLCnano with capillary flowmeter chromatographic systems (Thermo Fisher Scientific, San Jose, CA, USA). The analytical column was a C18 AQ capillary column with a 10 µm pulled tip (75 µm × 25 cm, 3 µm particle size; Columntip, New Haven, CT). A data dependent scan was used for initial identification of the erythrocyte frataxin N-terminal peptide (Acetyl-MNLRKSGTLGHPGSL). For peptide identification and quantification, the scheduled PRM was used instead.

### Data analysis

Data analysis for protein quantification was performed using Skyline (MacCoss Laboratory, University of Washington, Seattle, WA). The peak area ratio of each PRM transition for each unlabeled/light (L) peptide to labeled/heavy (H) peptide was calculated by the Skyline software and used for absolute quantification. The peptide ratios were calculated by the average L/H ratios of the three most intensive PRM transitions (Supplementary Table 1). The calculation of the frataxin levels was based on the L/H ratios using 1 and 20 ng frataxin standard protein as described previously^22^. Statistical analysis was performed using GraphPad Prism (v 5.01, GraphPad Software Inc., La Jolla, CA).

## Electronic supplementary material

Supplementary Information
