# Supplementary material for: Characterization of a new N-terminally acetylated extra-mitochondrial isoform of frataxin in human erythrocytes
Source: Sci Rep. 2018 Nov 19;8:17043. doi: 10.1038/s41598-018-35346-y (PMC6242848; doi:10.1038/s41598-018-35346-y)
Supplement: Supplementary file 1 — Supplementary Information [file 41598_2018_35346_MOESM1_ESM.pdf]

Supplementary Information

Characterization of a new N-terminally acetylated extra-mitochondrial isoform of frataxin in human erythrocytes

Authors: *Lili Guo,[1,2] Qingqing Wang,[1,2] Liwei Weng,[1] Lauren A. Hauser,[2,3,4] Cassandra J. Strawser,[2,3,4] Clementina Mesaros,[1,2] David R. Lynch,[2,3,4] and Ian A. Blair[1,2]*

[1]Penn SRP Center and Center of Excellence in Environmental Toxicology Center, Department of Systems Pharmacology and Translational Therapeutics Perelman School of Medicine, University of Pennsylvania Philadelphia, PA 19104, United States

[2]Penn/CHOP Center of Excellence in Friedreich's ataxia, Philadelphia, PA 19104, United States

[3]Departments of Pediatrics and Neurology, Children's Hospital of Philadelphia, Philadelphia, PA 19104, United States

[4]Departments of Pediatrics and Neurology Perelman School of Medicine, University of Pennsylvania, Philadelphia, PA 19104, United States

Supplementary Figures and Tables

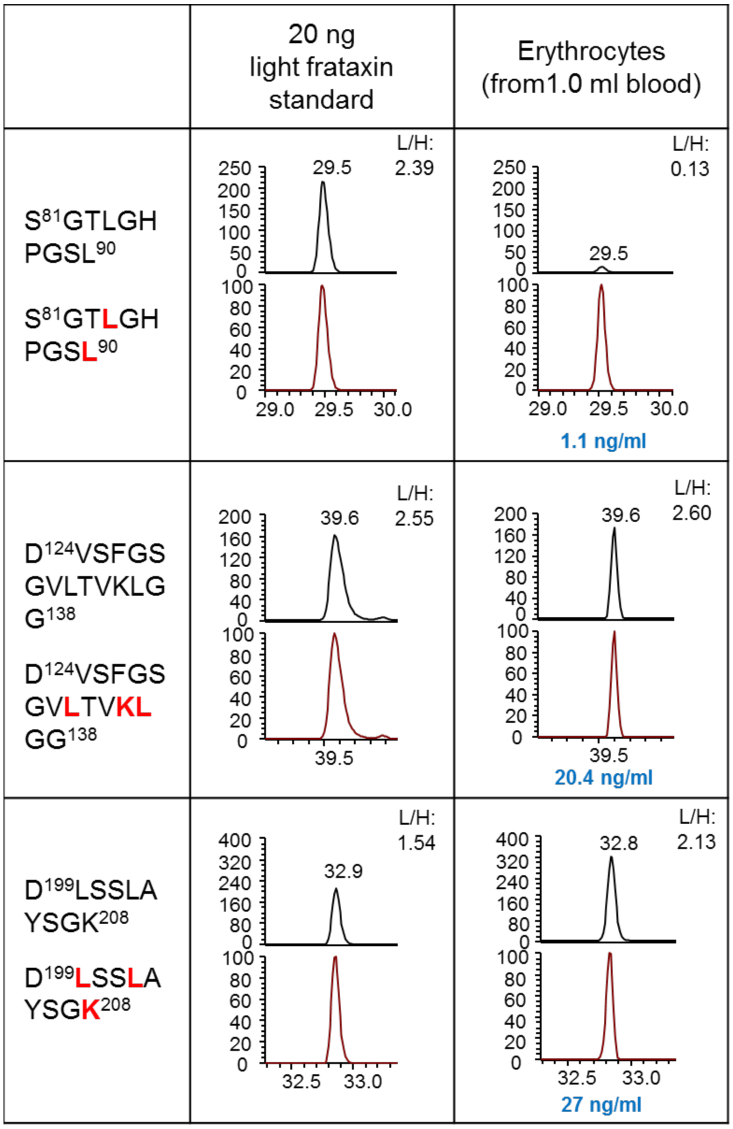

Supplementary Fig. 1 LC-MS chromatograms of AspN generated peptides from a standard mature frataxin (left) and erythrocyte frataxin. Black lines: endogenous frataxin; Red lines: SILAC frataxin standard. The labeled amino acids in each peptide are shown in red. Y-axis: relative abundance to the SILAC peptide signals. X-axis: time (min). The endogenous light peptide (L) and stable isotope labeled heavy peptide (H) peak area ratios based on three or four MS transitions (Supplementary Table 1) are shown. The blue text shows the calculated frataxin protein amounts calculated for the relevant peptide.

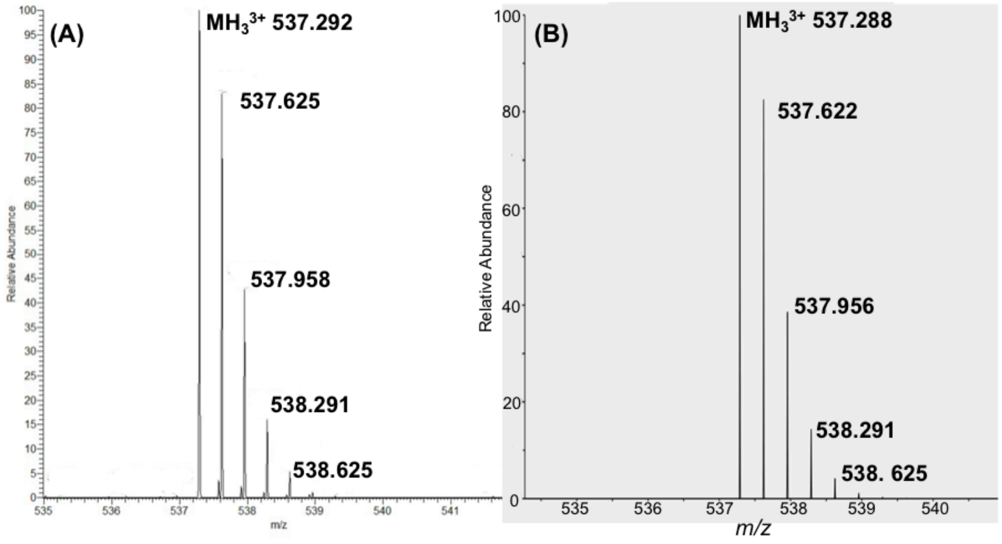

Supplementary Fig. 2 Mass spectra showing the triply charged protonated molecules from the first AspN peptide of erythrocyte frataxin: Ac-MNLRKSGTLGHPGSL. (a) Found. (b) Calculated.

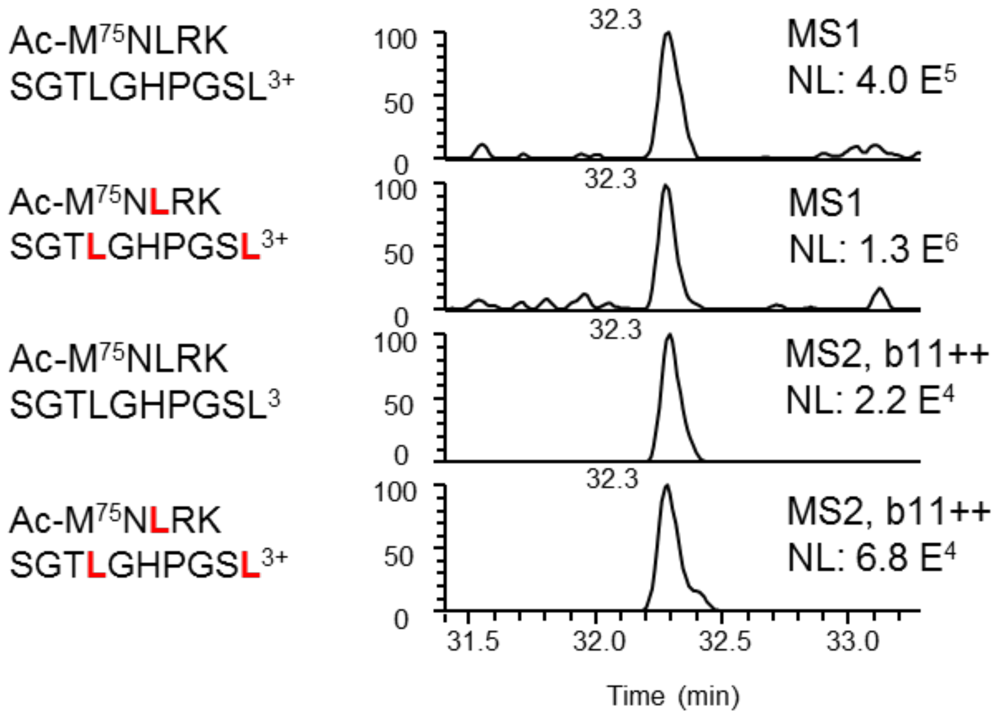

Supplementary Fig. 3 Chromatograms from LC-MS analysis of Ac-MNLRKSGTLGHPGSL. peptide generated from the erythrocyte frataxin and the synthesized AQUA isotope labeled heavy peptide spiked into the sample after AspN digestion. The heavy peptide was labeled by heavy with [$^{13}C_6{}^{15}N_1$]-leucine (red). Chromatograms from the endogenous AspN peptide and the heavy synthetic AQUA peptide are shown for full scanning (MS1) analysis (top two panels) and for one product ion ($b_{11}{}^{2+}$) from tandem MS analysis (bottom two panels).

	Precursor ion charge	Precursor ion (m/z)	Product ion	Product ion charge	Product ion (m/z)
**GluC digestion**					
Peptides used for quantification					
S81GTLGHPGSLDE	2+	585.2753	1+	y8	811.3581
			1+	y6	617.2777
			1+	b6	553.2729
			1+	b11	1022.4901
S81GT**L**GHPGS**L**DE	2+	592.2925	1+	y8	818.3752
			1+	y6	624.2949
			1+	b6	560.2901
			1+	b11	1036.5245
T93TYERLAEE	2+	556.2669	1+	y7	909.4312
			1+	y6	746.3679
			1+	b6	764.3937
			1+	b8	964.4734
T93TYER**L**AEE	2+	559.7755	1+	y7	916.4484
			1+	y6	753.3851
			1+	b6	842.448
			1+	b8	971.4906
L189TKALKTKLD	3+	377.5763	1+	y7	788.4876
			1+	y4	476.2715
			2+	y9	509.3188
			2+	y8	458.7949
**L189**T**K**A**LK**T**KL**D	3+	392.6077	1+	y7	818.5504
			1+	y4	491.3029
			2+	y9	528.3572
			2+	y8	477.8334
**AspN digestion**					
Peptides used for quantification					
S81GTLGHPGSL	2+	463.2405	1+	y7	680.3726
			1+	y6	567.2885
			1+	y4	373.2082
S81GT**L**GHPGS**L**	2+	470.2577	1+	y7	694.4069
			1+	y6	574.2057
			1+	y4	380.2253
D199LSSLAYSGK	2+	520.7666	1+	y6	638.3508
			1+	y5	525.2667
			1+	y4	454.2296
			1+	b3	316.1503
D199**L**SS**L**AYSG**K**	2+	531.7908	1+	y6	653.3822
			1+	y5	533.2809
			1+	y4	462.2438
			1+	b3	323.1675
D167WTGKNWVYSH	3+	464.8826	2+	y9	546.2671
			2+	y8	495.7432
			2+	y7	467.2325
D177WTG**K**NWVYSH	3+	467.554	2+	y9	550.2742
			2+	y8	499.7503
			2+	y7	471.2396
D124VSFGSGVLTVKLGG	2+	718.3932	1+	y11	987.5833
			1+	y7	687.44
			1+	y6	574.3559
			1+	y4	374.2398
D124VSFGSGV**L**TV**KL**GG	2+	729.4175	1+	y11	1009.6318
			1+	y7	709.4885
			1+	y6	589.3873
			1+	y4	389.2712
Peptides used for monitoring					
**Ac-M76**NLRKSGT LGHPGSL	2+	805.4276	1+	y4	373.2082
			2+	y14	718.9021
			2+	b11	619.3271
Ac-M76N**L**R**K**SGT **L**GHPGS**L**	2+	537.2875	1+	y4	373.2082
			2+	y14	718.9021
			2+	b11	619.3271

Supplementary Table 1: Peptides and transitions used for quantification of frataxin by LC-MS

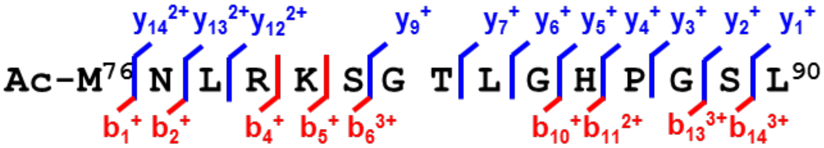

	Theoretical Mass	Determined Mass	Delta Mass (ppm)	Intensity
**$b1^{+}$**	174.0583	174.0571	-7	567098
**$b2^{+}$**	288.1013	288.1019	2.16	198744
**$b4^{+}$**	557.2865	557.2826	-6.96	111564
**$b5^{+}$**	685.3814	685.3769	-6.65	209701
**$b6^{2+}$**	258.1427	258.1390	-14.06	153781
**$b10^{+}$**	1100.5882	1100.5801	-7.3	266418
**$b11^{2+}$**	619.3272	619.3230	-6.67	2007034
**$b13^{3+}$**	464.5786	464.5749	-7.95	128366
**$b14^{3+}$**	493.5893	493.5856	-7.43	135795
**$y1^{+}$**	132.1019	132.1008	-1.09	256297
**$y2^{+}$**	219.1339	219.1324	-1.57	522460
**$y3^{+}$**	276.1554	276.1540	-1.45	161003
**$y4^{+}$**	373.2082	373.2055	-2.64	2043277
**$y6^{+}$**	567.2886	567.2840	-4.53	198439
**$y7^{+}$**	680.3726	781.4245	-6.15	156611
**$y9^{+}$**	838.4418	838.4282	-13.54	110173
**$y12^{2+}$**	605.3386	605.3430	4.36	2186955
**$y13^{2+}$**	661.8806	661.8774	-3.23	142502
**$y14^{2+}$**	718.9021	718.8967	-5.38	540337

Supplementary Table 2: List of b and y ions obtained from collision-induced dissociation of the triply charged protonated molecule (*m/z* 537.292) of endogenous erythrocyte frataxin first peptide Ac-MNLRKSGTLGHPGSL.
